# Transcriptomic differential lncRNA expression is involved in neuropathic pain in rat dorsal root ganglion after spared sciatic nerve injury

**DOI:** 10.1590/1414-431X20187113

**Published:** 2018-07-26

**Authors:** P. Mao, C.R. Li, S.Z. Zhang, Y. Zhang, B.T. Liu, B.F. Fan

**Affiliations:** 1 Department of Pain Medicine, China-Japan Friendship Hospital, Beijing, China; 2 State Key Laboratory of Toxicology and Medical Countermeasures, Department of Biochemical Pharmacology, Beijing Institute of Pharmacology and Toxicology, Beijing, China

**Keywords:** Spared nerve injury, Neuropathic pain, Long noncoding RNA, Transcriptome, *Hypm* gene

## Abstract

Dorsal root ganglia (DRG) neurons regenerate spontaneously after traumatic or surgical injury. Long noncoding RNAs (lncRNAs) are involved in various biological regulation processes. Conditions of lncRNAs in DRG neuron injury deserve to be further investigated. Transcriptomic analysis was performed by high-throughput Illumina HiSeq2500 sequencing to profile the differential genes in L4–L6 DRGs following rat sciatic nerve tying. A total of 1,228 genes were up-regulated and 1,415 down-regulated. By comparing to rat lncRNA database, 86 known and 26 novel lncRNA genes were found to be differential. The 86 known lncRNA genes modulated 866 target genes subject to gene ontology (GO) and KEGG enrichment analysis. The genes involved in the neurotransmitter status of neurons were downregulated and those involved in a neuronal regeneration were upregulated. Known lncRNA gene *rno-Cntnap2* was downregulated. There were 13 credible GO terms for the *rno-Cntnap2* gene, which had a putative function in cell component of voltage-gated potassium channel complex on the cell surface for neurites. In 26 novel lncRNA genes, 4 were related to 21 mRNA genes. A novel lncRNA gene *AC111653.1* improved *rno-Hypm* synthesizing huntingtin during sciatic nerve regeneration. Real time qPCR results attested the down-regulation of *rno-Cntnap* lncRNA gene and the upregulation of *AC111653.1* lncRNA gene. A total of 26 novel lncRNAs were found. Known lncRNA gene *rno-Cntnap2* and novel lncRNA *AC111653.1* were involved in neuropathic pain of DRGs after spared sciatic nerve injury. They contributed to peripheral nerve regeneration via the putative mechanisms.

## Introduction

After traumatic damage, the peripheral nervous system can regenerate spontaneously by activating the inherent growth ability of neurons, while the central nervous system cannot do so (1,2). The sciatic nerve is commonly used as a model to study peripheral nerve regeneration. It includes a complex bunch of motor and sensory axons, in which the sensory neurons are situated in the L4–L6 dorsal root ganglion (DRG) (3,4). After sciatic nerve damage, the damaged neurons initiate a regeneration process but cease having the neurotransmitter status (3,5). Axon regeneration and pathfinding after damage involves a complex mechanism involving axon cross-talk with neurogliocytes, nerve growth factors, neurotrophic factors, and receptors (6,7).

Neuropathic pain after traumatic or surgical nerve injury challenges doctors and patients and regulatory noncoding RNAs (ncRNAs) are key molecules for understanding and treating this pain (8,9). Regulatory ncRNAs are transcribed from protein noncoding genes to interfere in gene expression and they include, but are not limited to, miRNAs (21–24 nt), siRNAs (21–25 bp), piRNAs (26–31 nt), and long noncoding RNAs (lncRNAs, 200 bp to more than 100,000 bp) (8,10,11). The role of miRNAs, lncRNAs, and piRNAs in neuropathic pain after nerve injury has been reviewed by Bali and Kuner (8). The role and regulatory mechanisms of lncRNAs in vertebrate central nervous system and human nervous system diseases have been reported in the literature (8,12–15).

In peripheral nerve injury, lncRNAs play an important role in stress responses, plasticity, and axonal outgrowth of DRG neurons (8,16–21). Yu et al. (16) investigated the lncRNA transcriptome of DRG neurons after sciatic nerve injury in rat models and found that lncRNAs modulate DRG neurons responses to ligation stimuli. Zhao et al. (17) identified the modulating effect of the lncRNA (Kcna2 antisense RNA) on a voltage-dependent potassium channel mRNA Kcna2 in primary afferent neurons. Yao et al. (18) reported that the lncRNA uc.217 modulates neurite outgrowth of DRG neurons after sciatic nerve injury. lncRNA NONRATT021972 and lncRNA uc.48 modulate neuropathic pain mediated by the P2X(3/7) purinergic receptors (the cation-permeable ATP-binding ligand-gated ion channels) in the DRG neurons in diabetic rat models, and siRNA therapy alleviate the pain significantly (19–23). However, all these studies are based on microarray analysis (24). A sequencing study to elucidate vital roles of lncRNAs in peripheral nerve pathology will help to understand neuropathic pain.

In this study, we described the lncRNAs expression and mRNA expression in DRGs during nerve regeneration by a transcriptome-level deep sequencing. The results reveal a novel layer of regulation of the inherent growth ability of neurons by lncRNAs.

## Material and Methods

### Animal surgery and sample preparation

Six male Sprague-Dawley rats (180–220 g) were housed in large cages with sawdust bedding at 25°C in 12 h/12 h dark/light cycle and allowed free access to food and water in the Animal Center of Beijing China-Japan Friendship Hospital. Rats were randomly divided into test group and sham-operation group, three in each group. Surgery was performed as described in the literature with modifications (16). Briefly, rats were anesthetized by intraperitoneal injection of 10 wt.% chloral hydrate (3 mL/kg, Tianjin Fuchen Chemical Reagent Factory, China). The sciatic nerves were exposed and lifted through an incision on the right lateral thigh. Sciatic nerve segments were tied at the site proximal to the bifurcation of tibial and common peroneal nerves. Rats in the sham-operation group only had the sciatic nerves exposed without tying. L4–L6 DRGs were harvested from each animal a week later. All animal experiments were performed in accordance with institutional animal welfare and care guidelines and approved by the Animal Ethics Committee of Beijing China-Japan Friendship Hospital.

### RNA isolation, cDNA library preparation, and sequencing

Total RNAs were extracted from the L4–L6 DRG tissues using Trizol reagent according to the instructions of the manufacturer (Invitrogen, USA). RNAs were cleaned, including a DNase I digestion step, using RNeasy spin columns (Qiagen, USA). RNA integrity was detected by agarose gel electrophoresis and RNA was quantified using Nanodrop2000 (Bio-Rad, USA). After rRNA was removed, RNA was interrupted into short fragments by adding fragmentation buffer. These short RNA fragments were used as templates to synthesize the first-strand cDNA using FastQuant RT Kit (with gDNAase) (Tiangen, China). Then, double-strand cDNA was obtained. The cDNA products were purified with QiaQuick PCR extraction kit (Qiagen, Germany) and the purified cDNA were dissolved in EB buffer, followed by end reparation and poly(A) addition. The cDNA fragments were connected to sequencing adaptors. The cDNA fragments at 150–200 bp in size were separated on gel-electrophoresis and were used as the templates for PCR amplification. Two cDNA libraries for the test and sham-operation groups were sequenced using the Illumina HiSeq2500 at Beijing Ori-Gene Science and Technology Co., LTD (China).

### Data processing

Raw images generated by sequencers were converted by Illumina software (USA) to nucleotide sequences, called raw reads. FastQC software package (USA) was used to generate clean reads by removing adaptor reads, low quality reads (QC30), sequences containing fuzzy N bases and sequences less than 60 bp. All the following analyses were based on clean reads. The clean reads were mapped to the genome using Tophat2 software package (USA). RSeQC software package with all default parameters (USA) was used to detect the splice junction sites for evaluating the saturation of sequencing.

### Differential expression and enrichment

The RPKM method (reads per kilobase per million mapped reads) was used to calculate the differential expression level using the Cufflinks software package (USA). Cufflinks Cuffdiff was used to screen the differential expression genes (DEGs). The criterion to identify the DEGs between two groups was as follows: sum of mapping reads of two samples ≥10; |log2RPKM fold change| >1; P value was corrected by false discovery rate (FDR) to get a Q-value; both P≤0.05 and Q≤0.05 were required to determine the significance of differential expression. In order to get biological functions, DEGs were subjected to gene ontology (GO) and Kyoto Encyclopedia of Genes and Genomes (KEGG) enrichment analyses. R package was used for the analysis. GO terms of DEGs were compared with the genome background and the corrected P value (FDR correction) < 0.05 was set to judge the significantly enriched GO terms. For the KEGG pathway enrichment analysis, P value <0.05 was the threshold.

### Known and novel lncRNA

The RPKM method was used to calculate the known expression level using the Cufflinks software package. Because lncRNAs have no conservation between species, we used rat lncRNA database for annotating known lncRNA in the transcript obtained from sequencing. New transcripts with opening read frame features were aligned with known protein database with CPC scoring to predict either coding or non-coding RNA. The predicted non-coding RNA is a novel lncRNA.

### Coexpression network and target gene enrichment analysis

By comparing the differential gene lists, we obtained gene pairs of novel lncRNA and differential mRNA. FPKM values for each pair were used to calculate the Pearson correlation, where we chose significantly correlated gene pairs (correlation coefficient >0.995 or < –0.995, P<0.05 as threshold) to build a coexpression network using Cytoscape software package (USA). GO and KEGG enrichment analyses were performed as described above but the corrected P value threshold was set to <0.1. The correlated mRNA genes that coexpressed with lncRNAs served as the candidate target genes for lncRNAs.

### Real-time qPCR quantification

To quantify lncRNA and target mRNAs, real-time qPCR were performed on representative *rno-Cntnap2-201* lncRNA gene, *rno-Fam171b* mRNA gene, *rno-Hebp2* mRNA gene, *rno-Gde1* mRNA gene, *rno-AC111653.1* lncRNA gene, and *rno-Hypm* mRNA gene in the sham-operation and test DRG groups. Briefly, the first-strand cDNA was synthesized using FastQuant RT Kit (with gDNAase) (Tiangen). Then, double-strand cDNA was synthesized. Real time qPCR was performed on a Roche LightCycler¯ 96 fluorescence quantifying PCR machine. Primer sequences are listed in Table 1.

**Table 1.** Primer sequences.

Gene name	Sequence (5′ → 3′)
*rno-Cntnap2-201_F*	gcacctaccacaccaacga
*rno-Cntnap2-201_R*	tttgctctcgtcaatggtctct
*rno-Fam171b_5267F*	aggagttctgctttgctctgg
*rno-Fam171b_5460R*	tccacacacaaccaagggta
*rno-Hebp2_415F*	agatccgacactacggacca
*rno-Hebp2_662R*	cctgggtggatcatgttgct
*rno-Gde1_20F*	acgggtctggccgattatgt
*rno-Gde1_586R*	gcactctgtaactgcttccct
*rno-GAPDH_1096F*	gcccagcaaggatactgaga
*rno-GAPDH_1252R*	ggtattcgagagaagggaggg
*rno-AC111653.1_201F*	agctacagtcatggaaacaccc
*rno-AC111653.1_201R*	agatagcctcagctttgctcact
*rno-Hypm*_71F	cgacatgatg gtttgatggt
*rno-Hypm_251R*	ccatgcttga ttaccttacc

The reaction system included 1 µL of cDNA, 10 µL of RealStar Green Mixture (2×), 0.6 µL of primers, and filled up to 20 µL with PCR-grade water. The PCR program included a pre-denaturation at 95°C for 5 min, 40 cycles (95°C for 15 s, 60°C for 20 s, 72°C for 15 s) for amplification, and a default condition for dissociation. The cycle threshold (Ct) values were obtained. Relative interest/reference mRNA expression was calculated by the formula: 2-ΔCt (interest-reference). rno-GAPDH was used as inner reference.

### Cell assays

Wistar rat DRG cells were isolated and cultured as reported in the literature (25). Cell assays were performed as reported in the literature with a minor modification (26). Briefly, one-day-old Wistar rat DRG cells were isolated and cultured in 95% Eagle's DMEM feeding medium with 600 mg/mL glucose, 10% fetal bovine serum, 5% horse serum, 20 ng/mL nerve growth factors, and 1 μg/100 mL neurotrophins (25). For RNA silencing, siRNA sequences targeting lnc-*AC111653.1* were designed and synthetized (GenePharma, China), and a final concentration of 50 nM was used for transient transfection. For overexpression of lnc*-AC111653.1*, full-length rat lnc*-AC111653.1* cDNA was cloned into the pcDNA3.1 expression vector (GenePharma). Lipofectamine 3000 (Invitrogen) was used for transfection according to manufacturer's instructions (26).

### Western blotting

Total proteins were extracted from cell lysates and separated by 10% SDS-polyacrylamide gel. Then, they were electroblotted to PVDF membranes (Beyotime, China). Membranes were incubated in rabbit polyclonal HYPM antibody (Novus Biologicals, China), followed by horseradish peroxidase-labeled goat anti-rabbit secondary antibody (Boster, China). Signals were revealed using ECL detection reagent (Beyotime). GAPDH served as control. Images were analyzed by Image-Pro Plus 6 software (Media Cybernetics, USA). The intensity of test protein bands was normalized to the GAPDH bands.

## Results

### Gene mapping and differential expression genes

The RNA quality and sequencing quality were guaranteed. Clean reads were obtained. The results of gene mapping to the rat genome is shown in Table 2. Known gene expression is shown in Tables 3 and 4.

**Table 2.** Mapping results of transcriptome to referenced genome.

Sample	Total Reads (M)	Total Mapped (M)	Multiple Mapped (M)	Uniquely Mapped (M)
Sham 1	34,697	27,586 (79.50%)	7,890 (22.74%)	19,696 (56.76%)
Sham 2	31,648	25,293 (79.92%)	7,034 (22.23%)	18,258 (57.69%)
Sham 3	40,603	30,892 (76.08%)	9,391 (23.13%)	21,501 (52.95%)
DRG1	24,133	14,275 (59.15%)	6,434 (26.66%)	7,840 (32.49%)
DRG2	25,319	17,235 (68.07%)	7,139 (28.20%)	10,096 (39.88%)
DRG3	41,835	30,454 (72.80%)	10,614 (25.37%)	19,841 (47.43%)

DRG: dorsal root ganglia.

**Table 3.** Number and distribution of known gene expression.

Sample	Genes	Min.	1st Qu.	Median	Mean	3rd Qu.	Max.	Sd.	Sum.
Sham	17856	0	0.83	4.75	151.65	14.99	482618	5987.02	2707906
DRG	17296	0	1.15	5.15	>400.95	14.28	1828940	19976.57	6934761

DRG: dorsal root ganglia.

**Table 4.** Number and percentage of known gene expression.

Sample	0–0.5	>0.5–1	>1–5	>5–10	>10–50	>50
Sham	3634 (20.35%)	1184 (6.63%)	4322 (24%)	2764 (15.48%)	4488 (25.13%)	1464 (0.08%)
DRG	2859 (16.53%)	1175 (6.79%)	4504 (26%)	2996 (17.32%)	4416 (25.53%)	1346 (0.08%)

DRG: dorsal root ganglia.

**Figure 1.**
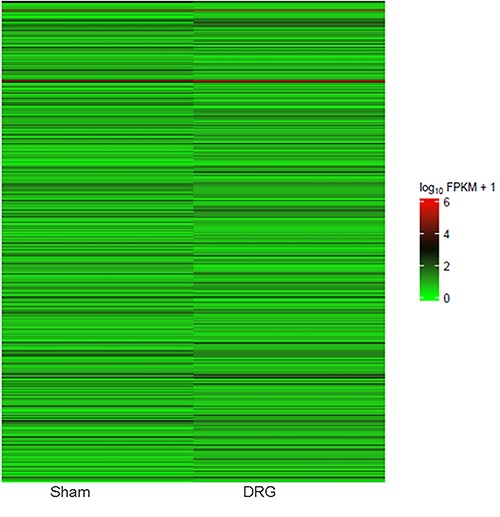
Genetic clustering of differential expression genes (threshold: fold-change >2). Red color represents high fold-change, green color represents low fold-change. Names were omitted due to many lines overlapping. DRG: dorsal root ganglia.

On differential expression analysis, a total of 18,824 genes were included, of which there were 2643 differential genes between DRG test group and sham-operation group. By comparison of the DRG group with the sham-operation group, 1228 were up-regulated and 1415 down-regulated. On enrichment analysis of DEGs, up-regulated differential genes were attributed to 624 GO terms and 50 KEGG pathways; down-regulated differential genes were attributed to 424 GO terms and 30 KEGG pathways. DEGs were clustered into a heatmap (Figure 1).

### Known lncRNA, co-expression network, and target gene enrichment

We found 69 neurite-associated known lncRNA genes linking to 866 target mRNA genes (Table 5). After the GO and KEGG enrichment information was presented at a P value threshold <0.1, the 866 targets were enriched to 737 GO terms and 40 KEGG pathways. They were involved either in the downregulation of neurotransmitter status of neurons or in the upregulation of peripheral neuronal regeneration. The GO terms and KEGG pathways involved in the downregulation effects included, but were not limited to, synaptic vesicle exocytosis, neurotransmitter secretion, voltage-gated potassium channel activity, regulation of synaptic transmission, GABAergic synapse, response to pain, endocytosis, neuronal action potential, detection of mechanical stimulus involved in sensory perception of pain, neurotransmitter transport, the GABAergic synapse pathway, the cholinergic synapse pathway, the neuroactive ligand-receptor interaction pathway, the dopaminergic synapse pathway, and the synaptic vesicle cycle pathway. The GO terms and KEGG pathways involved in the upregulation effects included, but were not limited to, response to mechanical stimulus, regulation of cell growth, positive regulation of cell migration, positive regulation of ERK1 and ERK2 cascade, positive regulation of PI3K signaling, activation of MAPKK activity, cell differentiation, regulation of neuron projection regeneration, regulation of nerve growth factor receptor activity, peripheral nervous system axon regeneration, glial cell differentiation, the AMPK signaling pathway, the calcium signaling pathway, the PI3K-Akt signaling pathway, the glucose metabolism pathway, the MAPK signaling pathway, and the cGMP-PKG signaling pathway.

**Table 5.** Known long non-coding RNA genes and their node degrees in Cytoscape co-expression network.

nodes_label	nodes_degree	nodes_label	nodes_degree	nodes_label	nodes_degree
ENSRNOG00000002734	47	ENSRNOG00000052027	3	ENSRNOG00000056599	4
ENSRNOG00000003025	47	ENSRNOG00000052373	1	ENSRNOG00000056608	2
ENSRNOG00000005811	1	ENSRNOG00000052439	3	ENSRNOG00000056656	1
ENSRNOG00000006617	43	ENSRNOG00000052563	37	ENSRNOG00000056824	3
ENSRNOG00000009373	1	ENSRNOG00000052573	2	ENSRNOG00000057161	12
ENSRNOG00000011160	45	ENSRNOG00000053160	2	ENSRNOG00000057278	13
ENSRNOG00000017974	4	ENSRNOG00000053367	6	ENSRNOG00000057291	1
ENSRNOG00000019648	122	ENSRNOG00000053827	1	ENSRNOG00000057463	1
ENSRNOG00000024799	55	ENSRNOG00000054418	2	ENSRNOG00000057991	1
ENSRNOG00000031706	4	ENSRNOG00000054489	1	ENSRNOG00000058263	1
ENSRNOG00000033581	88	ENSRNOG00000054529	2	ENSRNOG00000058571	12
ENSRNOG00000043199	13	ENSRNOG00000054533	5	ENSRNOG00000058935	2
ENSRNOG00000043866	5	ENSRNOG00000054867	3	ENSRNOG00000058944	3
ENSRNOG00000046171	21	ENSRNOG00000054897	3	ENSRNOG00000059449	3
ENSRNOG00000046774	3	ENSRNOG00000054935	1	ENSRNOG00000059660	1
ENSRNOG00000047117	1	ENSRNOG00000054984	1	ENSRNOG00000060090	6
ENSRNOG00000048929	31	ENSRNOG00000055021	2	ENSRNOG00000060430	2
ENSRNOG00000049537	12	ENSRNOG00000055067	1	ENSRNOG00000060483	1
ENSRNOG00000051356	2	ENSRNOG00000055278	2	ENSRNOG00000060700	1
ENSRNOG00000051492	13	ENSRNOG00000055939	42	ENSRNOG00000060863	64
ENSRNOG00000051664	29	ENSRNOG00000056040	7	ENSRNOG00000061151	2
ENSRNOG00000051722	3	ENSRNOG00000056054	5	ENSRNOG00000061536	3
ENSRNOG00000051924	3	ENSRNOG00000056490	1	ENSRNOG00000061622	1

**Figure 2.**
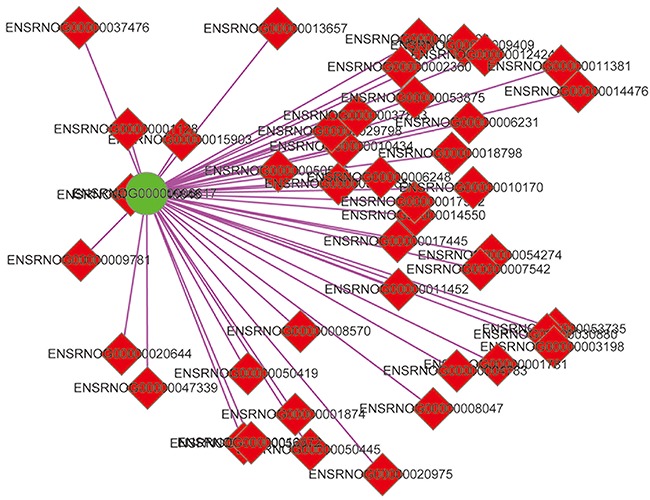
Co-expression network of gene *rno-Cntnap2* (*ENSRNOG00000006617*).

The target gene from GO enrichment of known lncRNA apparently pointed to *ENSRNOG00000006617* (P<0.05), thus we singled out the known lncRNA gene *ENSRNOG00000006617* named *rno-Cntnap2 (contactin associated protein-like 2*). Through serial analyses of molecular network (Figure 2) and GO enrichment on gene *rno-Cntnap2,* we found 13 credible GO terms at P<0.05 (Table 6).

**Table 6.** Gene Ontology (GO) terms of *rno-Cntnap2* long non-coding RNA gene.

Category	Term	Class	Gene_id
GO:0071205	protein localization to juxtaparanode region of axon	biological_process	ENSRNOG00000006617
GO:0044224	juxtaparanode region of axon	cellular_component	ENSRNOG00000006617
GO:0030673	axolemma	cellular_component	ENSRNOG00000006617
GO:0008076	voltage-gated potassium channel complex	cellular_component	ENSRNOG00000006617
GO:0019899	enzyme binding	molecular_function	ENSRNOG00000006617
GO:0043204	perikaryon	cellular_component	ENSRNOG00000006617
GO:0031175	neuron projection development	biological_process	ENSRNOG00000006617
GO:0005769	early endosome	cellular_component	ENSRNOG00000006617
GO:0007420	brain development	biological_process	ENSRNOG00000006617
GO:0030424	axon	cellular_component	ENSRNOG00000006617
GO:0030425	dendrite	cellular_component	ENSRNOG00000006617
GO:0043025	neuronal cell body	cellular_component	ENSRNOG00000006617
GO:0009986	cell surface	cellular_component	ENSRNOG00000006617

According to the 13 GO terms, *rno-Cntnap2* had a putative gene function that is involved in the cell component of voltage-gated potassium channel complex on cell surface of brain neurites where it has an enzyme binding activity. Considering the condition of the current study, it was assumed that *rno-Cntnap2* is involved in the cell component of voltage-gated potassium channel complex on cell surface of sciatic nerve neurites.

We reviewed the differential expression and co-expression network analysis of *rno-Cntnap2 (ENSRNOG00000006617)* gene, which was down-regulated (20.34 and 3.94 for sham group *vs* DRG group, fold change –2.37, P<0.001, Q=0.0003).

### Novel lncRNA, co-expression network, and target gene features

**Figure 3.**
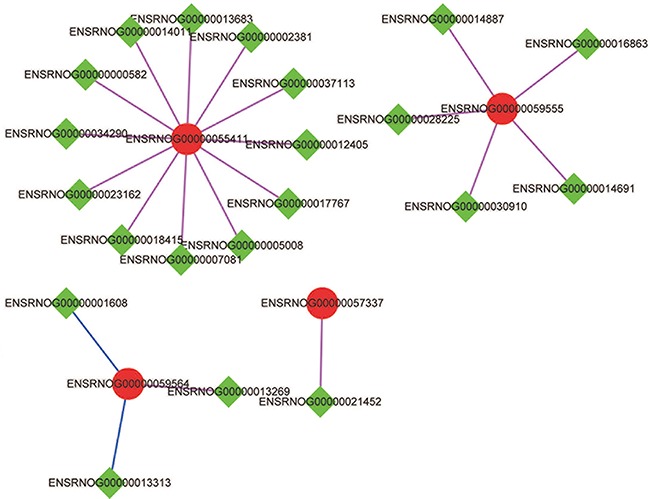
Co-expression network of novel long non-coding RNA (lncRNAs) with target mRNAs. The network comprises nodes and edges. Central round nodes are lncRNAs, green rectangle nodes are mRNAs. Purple edges indicate positive correlation and blue edges indicate negative correlation.

We found 525 novel transcripts containing 26 novel lncRNAs referenced to rat lncRNA database (Table 7). We constructed the co-network of novel lncRNAs with mRNAs, and only 4 lncRNAs were related to 21 mRNAs under the conditions of thresholds of <0.05 or 0.1 (Figure 3). The 4 lncRNAs were ENSRNOG00000055411, 00000059555, 00000059564 and 00000057337 (Table 7).

**Table 7.** Transcripts of 26 novel long non-coding RNA genes, of which 4 (in bold) are involved in co-expression network with target mRNA genes.

Transcript	Length bp	gene_id	Transcript	Length bp	gene_id
TCONS_00000233	509	ENSRNOG00000058258	TCONS_00016834	220	ENSRNOG00000036434
TCONS_00000914	1884	ENSRNOG00000058637	TCONS_00017920	149	ENSRNOG00000058995
TCONS_00001056	586	ENSRNOG00000056324	TCONS_00019224	1441	ENSRNOG00000000809
**TCONS_00002131**	**879**	**ENSRNOG00000055411**	TCONS_00021430	262	ENSRNOG00000053319
TCONS_00002609	194	ENSRNOG00000060612	TCONS_00021456	187	ENSRNOG00000047611
TCONS_00003909	1655	ENSRNOG00000054067	TCONS_00023970	2730	ENSRNOG00000056448
**TCONS_00009651**	**593**	**ENSRNOG00000059555**	TCONS_00028110	255	ENSRNOG00000036492
TCONS_00011039	350	ENSRNOG00000061204	TCONS_00028111	217	ENSRNOG00000040358
TCONS_00012047	330	ENSRNOG00000035462	TCONS_00028926	281	ENSRNOG00000047126
TCONS_00012411	134	ENSRNOG00000052738	TCONS_00033369	1000	ENSRNOG00000057258
**TCONS_00014951**	**153**	**ENSRNOG00000059564**	TCONS_00034793	312	ENSRNOG00000032609
TCONS_00015936	235	ENSRNOG00000035501	TCONS_00035961	1447	ENSRNOG00000051245
**TCONS_00016823**	**828**	**ENSRNOG00000057337**	TCONS_00036296	341	ENSRNOG00000035579

We noticed that the transcript TCONS_00016823 included only one novel lncRNA gene, *AC111653.1* (*ENSRNOG00000057337*), with a sense strand length of 828 nt. Gene *AC111653.1* was null expressed in the sham-operation group and upregulated to 0.527889 in the DRG group. Gene *AC111653.1* was correlated to the target gene *ENSRNOG00000021452* named *huntingtin interacting protein M (rno-Hypm).* The *rno-Hypm* gene GO annotations included the molecular function of DNA binding and protein heterodimerization activity, the biological process of chromatin silencing, and the cellular component of nuclear chromatin and nucleosome. Huntingtin is essential for neuron survival, and the lack of huntingtin synthesis may lead to Huntington's disease (27). Up-regulation of both *AC111653.1* and *rno-Hypm* genes after sciatic nerve injury implies a rescue course that triggers the regeneration of injured neurons. However, the function of *Hypm* gene is not completely understood.

### Quantification of several genes

For known lncRNAs detection, we selected *rno-Cntnap2* lncRNA gene and three down-regulated gene representatives, *Fam171b* (*ENSRNOG00000004783*), *Hebp2* (*ENSRNOG00000053735*), and *Gde1* (*ENSRNOG00000050445*) from the *rno-Cntnap2* gene coexpression network (Figure 2). Real time qPCR was performed to quantify expression levels of the four genes. The quantification results are shown in Table 8. Expression levels of the four genes were down-regulated. This result was consistent with the sequencing outcomes.

**Table 8.** Real time qPCR quantification of interest/GAPDH (interest DRG/Sham) gene expression levels.

	Sham	DRG1	DRG2	DRG3	Sequencing (Sham/DRG)
*rno-Cntnap2-20 lncRNA*	0.0530 (1.0000)	0.0074 (0.1411)	0.0069 (0.1310)	0.0039 (0.0736)	20.3371/3.9422
*rno-Fam171b*	0.0265 (1.0000)	0.0053 (0.1993)	0.0012 (0.0446)	0.0026 (0.0981)	19.7562/6.0137
*rno-Hebp2*	0.0417 (1.0000)	0.0112 (0.2673)	0.0145 (0.3475)	0.0082 (0.1966)	97.8422/22.9642
*rno-Gde1*	0.0128 (1.0000)	0.0097 (0.7526)	0.0042 (0.3231)	0.0065 (0.5078)	93.1178/36.7535
*rno-AC111653.1 lncRNA*	0	0.08652	0.04228	0.03684	0/0.52789
*rno-Hypm*	0	0.29365	0.1263	0.1046	0/2.6653

DRG: dorsal root ganglia. The numbers outside parenthesis indicate the gene expression ratio of interest to GAPDH and those inside indicate the gene expression ratio of interest DRG groups to the Sham group.

For novel lncRNAs identification, we selected *AC111653.1* gene (*ENSRNOG00000057337*) and *rno-Hypm* gene (*ENSRNOG00000021452*) from the *AC111653.1* gene coexpression network (Figure 3). They had a correlation coefficient of 1 (significance P=0). Up-regulation of both AC111653.1 and *rno-Hypm* genes after sciatic nerve injury may imply a rescue course that triggers the regeneration of injured neurons, thus the function of *Hypm* gene deserved to be studied. Real time qPCR was performed to quantify expression levels of the two genes (Table 8), which were up-regulated. This result was consistent with the sequencing outcomes.

### Cell assays

**Figure 4.**
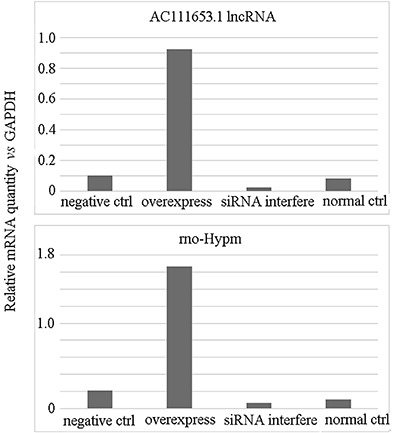
QPCR relative mRNA quantification. Upper panel shows rno*-*lncRNA AC111653.1 levels. Lower panel shows rno-Hypm mRNA levels in each group. Negative ctrl: pcDNA3.1 vector transfection; overexpress: pcDNA3.1-lnc-AC111653.1 transfection; siRNA interfere: siRNA-lnc-AC111653.1 transfection; normal ctrl: normal cells without treatment.

**Figure 5.**
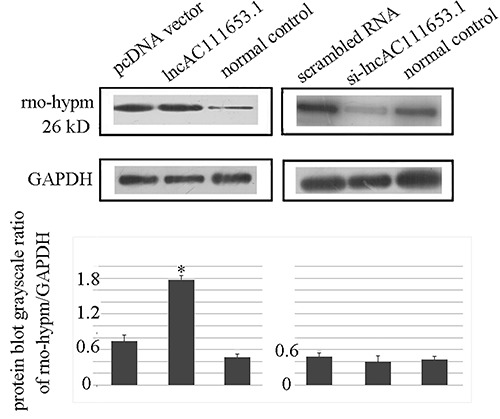
Western blot images. Cells were transfected with pcDNA3.1-lnc-AC111653.1 for overexpression and with siRNA-lnc-AC111653.1 for RNA interference. pcDNA3.1 vector and scrambled RNA served as negative controls. *P<0.05 compared to the other groups (ANOVA).

To test the biological function of novel lncRNA *AC111653.1* gene, we detected *AC111653.1* and its target Hypm in primarily cultured Norway rat DRG cells *in vitro*. QPCR results are shown in Figure 4, and western blots in Figure 5. Novel lncRNA *AC111653.1* was overexpressed after pcDNA3.1-lnc-AC111653.1 transfection. At the same time, its target hypm was also upregulated. Expression of AC111653.1 was reduced after siRNA transfection, and at the same time, its target hypm was downregulated. This suggested that novel lncRNA AC111653.1 was positively associated with hypm regulation in rats.

## Discussion

In this study, we used a common sciatic nerve injury model to investigate gene expression conditions in rat DRGs using a high-throughput Illumina HiSeq2500 sequencing. In total, 86 known lncRNAs and 26 novel lncRNAs were altered during nerve regeneration. To understand the functions of the 86 known lncRNAs, we analyzed the molecular network including 866 co-expressed target genes. After sciatic nerve damage, the nerve systems switched from a neurotransmitter status to a neuronal regeneration status (1,3,5).

Based on the GO and KEGG enrichment results, we found that the neurotransmitter status of neurons are deregulated by the molecular mechanisms linking to the deregulation of the neuroactive neurotransmitter secretion, transmission, and ligand-receptor interaction pathway, while the neuronal regeneration was activated through the molecular mechanisms linking to the positive regulation of cell migration, cell differentiation, cell growth, PI3K signaling, MAPK cascade activity, nerve growth factor receptor activity, and peripheral nerve regeneration. Glial cells migration, dedifferentiation, differentiation, proliferation, and growth play important roles in peripheral nerve regeneration (1–4). The results in this study showed the promotion of glial cell migration and growth by multiple signaling pathways. After sciatic nerve damage, local Schwann cells can shed off the myelin sheaths and transform to a neuroblast status, where their proliferation and migration capacities can help to sweep away myelin remnants and generate a conduit for the axonal pathfinding, and consequently form the beneficial conditions for neurite outgrowth (1–3). The same lncRNA-linked nerve regeneration mechanism is identified by Yu et al. (16) and Yao et al. (18).

We singled out the known *rno-Cntnap2* lncRNA gene, thought to be involved in the cell component of voltage-gated potassium channel complex on cell surface for the neurites of the sciatic nerve system. We speculated that sciatic injury might trigger a switch from a neurotransmitter status to a regeneration status of neurons. The gene *rno-Cntnap2* may be involved in a neurotransmitter delivery process linking to the function of voltage-gated potassium channel complex. Thus, *rno-Cntnap2* gene expression was down-regulated because a neurotransmitter status was ceased. The modulation of voltage-gated potassium channels by a lncRNA has been identified in DRG first-order sensory neurons in a spinal nerve ligation rat model (17). In this previous study, peripheral nerve injury increased a conserved lncRNA (Kcna2 antisense RNA) expression in injured DRG through activation of the transcription factor myeloid zinc finger protein 1. This increase of lncRNA downregulates the voltage-dependent potassium channel Kcna2 mRNA, consequently reducing total potassium current. The decrease of potassium current increases the neural excitability, namely neuropathy-induced sensitivity to mechanical stimuli in DRG neurons, resulting in neuropathic pain symptoms. The modulation of rno-*Cntnap2* mRNA may also follow this molecular mechanism, though identification is required.

We further selected the transcript TCONS_00016823 containing only one novel lncRNA gene *AC111653.1* (*ENSRNOG0000005733*). This lncRNA's upregulation improved the *rno-Hypm* gene expression, which promoted huntingtin synthesis regenerating sciatic nerves. We tested the biological function of novel lncRNA *AC111653.1* in rat dorsal root ganglion cells. The overexpression of lncRNA *AC111653.1* upregulated *rno-Hypm* gene substantially, indicating that this novel lncRNA is accurately associated with the huntingtin protein regulation.

The time-course factor should be considered a limitation because transcript levels vary depending on the time between the mechanic stimuli of nerve tying until the detection starts (16). Thus, time-dependent gene expression change and more testing on lncRNA functions should be done in the future. In addition, more annotations on genes should be investigated.

In conclusion, a total of 26 novel lncRNAs were found. Both down-regulated *rno-Cntnap2* gene and up-regulated *rno-Hypm* gene were involved in neuropathic pain of DRGs after spared sciatic nerve injury, thus contributing to peripheral nerve regeneration via putative mechanisms.
